# Micro‐provision of Social Care Support for Marginalized Communities – Filling the Gap or Building Bridges to the Mainstream?

**DOI:** 10.1111/spol.12114

**Published:** 2015-03-05

**Authors:** Catherine Needham, Sarah Carr

**Affiliations:** ^1^ University of BirminghamBirminghamUK

**Keywords:** Social care, Personalization, Micro‐enterprise, Seldom heard, Black and minority ethnic, Lesbian, gay, bisexual and transgender

## Abstract

As English social care services reconstruct themselves in response to the personalization agenda, there is increased interest in the contribution of micro‐providers – very small community‐based organizations, which can work directly with individuals. These micro‐providers are assumed to be able to cater for the ‘seldom heard’ groups which have been marginalized within mainstream social care services. This article reviews recent literature from the UK published in peer‐reviewed journals from 2000 to 2013 on support provision for people with protected characteristics under the Equality Act 2010. It considers the marginalising dynamics in mainstream, statutory social care support provision, and how far local community, specialist or small‐scale services are responding to unmet need for support and advice among marginalized groups. The review found that there is a tradition of compensatory self‐organization, use of informal networks and a mobilization of social capital for all these groups in response to marginalization from mainstream, statutory services. This requires recognition and nurturing in ways that do not stifle its unique nature. Specialist and community‐based micro‐providers can contribute to a wider range of choices for people who feel larger, mainstream services are not suitable or accessible. However, the types of compensatory activity identified in the research need recognition and investment, and its existence does not imply that the mainstream should not address marginalization.

## Introduction

As English social care services reconstruct themselves in response to the personalization agenda, there is increased interest in the idea that local authorities will become market‐shapers rather than providers or even commissioners of services. Direct payments, and to a lesser extent, managed personal budgets, can be a mechanism through which individuals themselves commission support. In this landscape of personalized care and support, there will be a key role for micro‐providers – very small community‐based organizations, which can work directly with individuals. Such enterprises are expected to take an increased share of the adult social care market in the future (DH 2010). These enterprises are a realization of the government plans to develop the world's largest social enterprise sector as part of a ‘Big Society’ (Needham 2011; Ishkanian and Szreter 2012). The Department of Health's (DH's) enthusiasm for micro‐enterprise appears to stem from a common sense assumption that small organizations are ‘closer to the user’, and therefore more responsive, than larger ones (DH 2010). A departmental guidance document on micro‐enterprise asserts, ‘Micro‐social care and support enterprises established and managed by local people are in a good position to deliver individualised services and are vital elements of a diverse market’ (DH 2010).

The term ‘micro‐provider’ typically refers to organizations employing five staff or fewer – although, like social enterprise, it lacks a fixed definition (MacGillivray *et al*. 2001; Fiedler 2007; Pattie and Johnston 2011). They are commonly set up to meet the needs of an individual or small group (Shared Lives Plus n.d.). One of the expectations of micro‐providers is that they will be able to cater for the ‘seldom heard’ groups which have been marginalized within mainstream social care services. Adult social care works with some of the most disadvantaged people in society, and social work has a tradition of inclusion, empowerment and anti‐oppressive practice (Dominelli 2010). However, the reality is that a number of seldom heard groups experience aspects of mainstream, traditional social care provision as inaccessible or disempowering. In terms of achieving the necessary flexibility and responsiveness, community‐based micro‐provision could be a particularly appropriate option for configuring social care support.

This article reviews recent literature from the UK published in peer‐reviewed journals from 2000 to 2013 on support provision for people with protected characteristics under the Equality Act 2010, who can be seldom heard in mainstream services. It considers the marginalizing dynamics in mainstream, statutory social care support provision, and how far local community, specialist or small‐scale services are responding to unmet need for support and advice among marginalized groups. The first section looks at the context of social care reform in England. The second section considers the lack of support for groups with protected characteristics in mainstream services. The third section sets out how self‐organization has developed to fill this gap. The fourth section assesses the intersection between mainstream support and self‐organization, recognizing that they often interact. The conclusion brings these findings together and considers how local authority commissioners and market‐shapers can most effectively support self‐organization without abdicating responsibility to marginalized groups.

## The Policy Context

Recent social care policy and implementation strategies as outlined in *A Vision for Adult Social Care: Capable Communities, Active Citizens* (DH 2010) and in the Think Local Act Personal (TLAP) partnership agreement (TLAP 2011), emphasize that increasing choice and control and building community capacity are interrelated personalization policy objectives. The National Care Forum, part of the TLAP partnership, has argued the case for the distinctive contribution that local not‐for‐profit support providers can make to added value and social capital in social care, as well as improving choice (IPC 2012). Community Catalysts, an organization which provides practical support and advice to micro‐providers, has argued that people who have experienced care and support services are well placed to ‘spot any gaps in services and supports within their community and could be well placed to fill these gaps’ (Community Catalysts 2011: 3). Thousands of micro‐providers exist in the adult care sector, purporting to offer a mid‐point between inflexible block commissioning by local authorities and *ad hoc* purchasing by personal budget holders which may leave some users unsupported (DH 2010). Governance models are varied and include charities, sole traders, partnerships, community interest companies and mutuals. Some are set up by frontline staff previously based in large organizations, whereas others are created by people who need support and/or carers. They may provide core ‘care’ services (e.g. day support services; domiciliary care; residential care), or have a broader focus (e.g. leisure, therapies, employment) (DH 2010).

In their baseline survey of micro‐providers, Bull and Ashton (2011) found that the majority offered specialist support and were established to help people and communities in a local area. Dickinson *et al*. (2012) argue that new types of social care support provision, such as micro‐providers and small community social enterprises, should increase as a result of personalization. Such local support is to scale and can have the flexibility, responsiveness and quality of relationships required by people who use social care and support. In terms of building community capacity, such ‘bottom–up’ developments can draw on unique cultural intelligence, building on individual and collective assets, knowledge, networks and strengths (Dickinson *et al*. 2012: 28).

Adult health and social care reforms are occurring within the wider equality and diversity policy framework, with the Equality Act 2010 underpinning public policy developments and defining those with ‘protected characteristics’ under the Act. The Health and Social Care Act 2012 legislates for the duty to reduce inequalities in health and social care. Research continues to show that people from particular minority groups or those with ‘protected characteristics’, such as black and minority ethnic (BME) people or lesbian, gay and bisexual (LGB) people, remain marginalized by mainstream services in social care and mental health (Chahal 2004; Ward *et al*. 2010). It has been argued that by using personal budgets and direct payments, people who are marginalized by the mainstream can purchase culturally appropriate support and improve choice in local social care markets (Voice 4 Change 2012). Some local authorities with a high density populations of BME communities or LGB people have made efforts to engage with those communities to understand their needs and support networks better (Carr 2013). In this policy context, the necessary flexibility and responsiveness could be achieved by further developing community‐based micro‐provision as a social care and support choice.

## Methods and Scope

The research review aims to identify support provision for people with protected characteristics under the Equality Act 2010. It considers the marginalizing dynamics in mainstream, statutory social care support provision, and how far local community, specialist or small‐scale services are responding to unmet need for support and advice among marginalized groups. While it is not a systematic review, it has been systematically conducted and is informed by Greenhalgh's and Peacock's (2005) model of searching for complex evidence. The methodology is guided by the ‘narrative review’ approach and the studies included have been subject to thematic analysis.

Searches were conducted for empirical research or research review (including systematic review) papers focusing on the UK which were published in English in peer‐reviewed journals about social care service provision for working age adults and older people, including carers, from 2000 to 2013. The databases searched were the Health Management Information Consortium (HMIC), Proquest, EBSCO, Cinahl, Social Science Citation Index, Social Services Abstracts, ASSIA, Social Care Online and Google Scholar. Cochrane and Campbell collaboration reviews were also searched. The groups or topics covered in the search strategies were: BME people; lesbian, gay, bisexual and transgender people; older people; carers; religion and belief; refugees and asylum seekers. Inclusion and exclusion criteria were used to screen and select the final includes for the review. Studies were included if the abstracts indicated that they focused on community‐based (not residential or nursing home) social care provision (including information and advice, advocacy, safeguarding; excluding health, workforce issues) for working age adults (excluding children, families or youth) and older people in the UK. The abstracts developed by the initial searches were assessed by the authors, with a full reading of any studies over which there was disagreement about inclusion. The studies which passed this stage were then read in full by one of the authors and any that did not meet the criteria above were removed.

Eighty‐five papers were initially identified and after screening, 45 peer‐reviewed journal research papers were finally included. The research evidence on the experience and effectiveness of local community, specialist or small‐scale services is of varying type and quality. The majority of the studies used qualitative and case study methods, but there were some papers presenting small‐scale surveys (including cross‐sectional surveys) and existing data set analysis. The number of participants in interview‐based qualitative studies ranged from six to 100. There were a number of evaluations of community‐based, local or specialist support services and of mainstream service practice that were variable in quality. The rest of the included papers were topic‐relevant narrative accounts, brief research overviews with expert commentary and practice commentaries. No systematic reviews or randomized controlled trials were identified in the searches.

The majority of the included studies looked at issues and experiences of BME communities, with a large number of papers dedicated to understanding the role of family carers, particularly from South Asian backgrounds. Other BME groups covered in the research included Irish people, Somali people, Ethiopian people and Chinese people. No study was found specifically on Black African or Caribbean people in the UK, although findings for this group appear in several of the generic BME research reviews. A smaller body of work on LGB older people and carers was found. Similarly, a number of research studies on support for and by refugees and asylum seekers were identified. Some research on the role of faith was also found. Lastly, a number of evaluations for specific initiatives were identified and included. Because of their particular relevance to the review, and contextual similarity to the UK, one study from Australia and two studies from Ireland were included. The search exercise suggests that there are gaps in the UK research published in peer‐reviewed journals in English for gypsies and travellers, transgender people and for people who might experience multiple discrimination such as LGB people from BME communities.

## Limitations of Mainstream Support

A consistent finding across all groups was a perception or fear of mainstream, traditional care and support as discriminatory. In his comparative study of the expectations of support among White British and Asian‐Indian older people in Britain, Sin (2006: 216) notes that, ‘a person's perception of the adequacy or quality of support is inevitably influenced by his or her expectations of the type, frequency and source of support preferred or required’. This was true for BME communities, LGB people, people from certain faith groups and asylum seekers and refugees, as the research studies suggested that all feared discrimination or misunderstanding, had low expectations of the suitability or accessibility of support and even feared interventions from large, generalist or mainstream providers (Price 2012, 2010; Mir and Tovey 2003; Sin 2006; Yeung and Ng 2010; Cronin *et al*. 2011; Papadopoulous *et al*. 2004; Heaphy *et al*. 2003; Williams 2006; Chau and Yu 2009).

This perception or experience of the mainstream led some LGB people and carers from BME communities to avoid using traditional services in order to maintain control over their lives and identities or to avoid stress and feelings of powerlessness (Mir and Tovey 2003; Sin 2006; Price 2012, 2010). A study on South Asian parents of adult children with cerebral palsy found that, ‘feelings of inability to influence or control the response from services led parents to avoid initiating contact’ (Mir and Tovey 2003: 472). Similarly, a study of the LGB carers of LGB people living with dementia showed anxiety and stress in accessing mainstream support particularly because ‘privacy was something all respondents valued and strived to maintain and many people feared for a future where such control was no longer possible’ (Price 2012: 523).

The studies suggested some reasons for the perception or experience of discrimination and misunderstanding in mainstream, traditional services which can lead to non‐engagement or disengagement. Again, for both BME communities and LGB people (and in some cases for refugees and asylum seekers) there were consistent findings around four themes: first, homogenization and diversity blindness; second, language and communication; third, the role of friends and family; and, fourth, the role of faith.

On the first of these themes, a study on the consequences of using administrative categories leading to homogenization for LGB older people suggested that it is important to, ‘recognise the fact that LGB people do not simply constitute one easily defined, socially homogenous group whose needs are similar simply by virtue of membership’ (Cronin *et al*. 2011: 425). Likewise, stereotyping and homogenization was identified as a difficulty for Chinese people when engaging with mainstream social care and support, ‘Analysts warn that over‐generalization in terms of racial or ethnic group characteristics may undermine our understanding of culturally diverse groups’ (Chau and Yu 2009: 775).

Diversity blindness and universalism limited the degree to which mainstream support could respond in a culturally sensitive way or could accommodate the particular support needs of the individual, particularly in the context of personalization in adult social care (Cronin *et al*. 2011; Batsleer *et al*. 2003; Chau and Yu 2009; TLAP 2011). A study concerning the responses of health and social care staff to South Asian women who self‐harm or who have attempted suicide, identified several types of ‘neutrality’ in practice as having an impact on the quality and accessibility of support, ‘First there is the “race neutral” or universalist approach. The second approach can be termed the “gender neutral” approach, in which issues of “race” and culture are privileged over all other issues in the form of supposedly “ethnosensitive’ services”’ (Batsleer *et al*. 2003: 109). An additional dimension to diversity blindness or neutrality can be identified for older LGB people where ‘sexuality blindness’ leads to invisibility and difficulties around feeling safe and ‘coming out’ (Price 2010). Also focusing on LGB older people, Cronin *et al*. (2011: 424) make an important point about neutrality and equality in mainstream social care, ‘a … “blind” approach, although reasoned by service providers as equal treatment, [is] somewhat difficult to justify’, particularly in the context of personalization and the Equality Act 2010 (Ward *et al*. 2010).

A second theme, language and communication, is a source of enduring difficulties for people from BME communities and for refugees and asylum seekers when accessing mainstream social care services (Moriarty 2008; MacFarlane *et al*. 2009; Merrell *et al*. 2006). The included studies showed that language barriers remain a major concern for those who are not fluent in English and that interpretation methods are not always effective (MacFarlane *et al*. 2009; Merrell *et al*. 2006). One study of language barriers in social care and interpretation services indicated the need for sensitivity about confidentiality, trust and anonymity if using community‐based interpretation services, ‘while strong local networks have advantages for well‐being in terms of social capital, there may be disadvantages in terms of a “goldfish bowl” effect’ (MacFarlane *et al*. 2009: 209). For older LGB people, communication difficulties have been associated with disclosing personal history and identity, especially for LGB people living with dementia and for their carers (Price 2012, 2010; Cronin *et al*. 2011).

A third theme was mainstream assumptions about family and friendship caring patterns, particularly for South Asian people who can be caricatured as completely self‐reliant. For example, a study on South Asian family caring found that the main family carer (usually female) did not necessarily have access to extended family support, concluding that, ‘the findings challenge the pervasive assumption and stereotype that South Asian people live in self‐supporting extended families, and therefore, that the support of social services is largely unnecessary’ (Katbamna *et al*. 2004: 404). Research into Bangladeshi carers also found that misunderstanding and cultural stereotyping contributed to ‘differential practice being delivered’ and that there was a lack of appropriate outreach (Merrell *et al*. 2006). Another study suggested that, ‘most Asian‐Indian families tend to see state support as a poor substitute for family support’ (Sin 2006: 220). Based on their research findings, Victor *et al*. (2011: 92) recommend that, ‘social care‐based services may be more appropriate and acceptable if they focus on helping and supporting families to care rather than being viewed as substitutes for family care’. Several studies showed complex, global webs of family relationships or ‘superfamilies’ (Sin 2006) for certain BME communities and for refugees and asylum seekers, also with particular implications for support and advice services (Victor *et al*. 2011).

For LGB people, the role of friends (as well as same‐sex partners) is a key element of care and support (White and Cant 2003; Heaphy *et al*. 2003). Again, the research shows that informal support for older LGB people can be subject to assumptions and stereotyping by mainstream services and can effect carer engagement, confidence and help‐seeking behaviour (Price 2012, 2010). A study on support networks of HIV‐positive gay men found that, ‘partners, ex‐partners and friends were likely to be seen more frequently than family members, and therefore, are more able to offer daily emotional and instrumental support’ (White and Cant 2003: 331). The same was found for older LGB people, where ‘friends are on a par with partners and family in terms of material support in times of need’ and ‘few expect family members to assume this [care and support] responsibility’ (Heaphy *et al*. 2003: 33). The pressure on partner carers of older LGB people with dementia to ‘come out’, disclose or not hide their same‐sex relationship status to mainstream services was found in several research studies to be a source of anxiety and in some cases discrimination, thereby resulting in mainstream support being inaccessible or of poor quality (Price 2012, 2010). One study concluded that a key difficulty lay in ‘how caring … [is] framed in accordance with heteronormative social relations’ (Cronin *et al*. 2011: 427).

A fourth theme encompassed cultural and faith issues in a context of social care support. A number of studies raised the cultural issue of stigma and shame among some BME communities and among refugees and asylum seekers, requiring particular awareness and sensitivity and influencing mainstream service use. For BME older people, ‘the existence of stigma, particularly about mental health problems in old age, may be higher in some communities than in others’ (Moriarty 2008: 3). The issue of confidentiality and mental health stigma was found in research on orthodox Jewish people in the UK, South Asian people, Irish older people and for Somali older men (Loewenthal 2012; Silveira and Allebeck 2001; Cant and Taket 2005). Yeung and Ng (2010: 294) conclude that, ‘the cultural issues about shame, losing face and other traditional Chinese beliefs … need to be thought through carefully when planning [for] this marginalised community’. Further complexities around mental health, stigma and gender were found for South‐Asian women (Batsleer *et al*. 2003). Stigma and shame were found to arise in relation to ‘illness’ and to formal service use for some BME families (Sin 2006), as Victor *et al*. (2011: 90) note for carers from South Asian communities in the UK, ‘having to turn to the state for care was clearly construed extremely negative … indicating lack of family loyalty and potential loss of face within the wider community’. This finding also appeared in Loewenthal's (2012) study of orthodox Jewish people and mental health support.

Research into the experiences of Bangladeshi carers suggested that they were sometimes reluctant to seek help for personal care from mainstream, traditional sources because there was a perception that these services would be ‘unable to meet their cultural and religious needs’ (Merrell *et al*. 2006: 203). Similar conclusions were reached for other BME communities and for LGB people (Moriarty 2008; Price 2012, 2010). Evidence suggests that faith and culture are important for resilience and well‐being. Networks of initiatives which can encourage this type of support should be recognized as an important source of ‘social capital’ and an asset. However, research also shows complexity and tension when it comes to faith and religion in support services (Hopkins 2011; Daley 2007). Simplistic approaches to religion and faith or assumptions that faith‐based projects can unproblematically provide support are unhelpful (Loewenthal 2012; Chau and Yu 2009; Batsleer *et al*. 2003; Furness and Gilligan 2010). For example, for South Asian women's mental health ‘misinformed ideas about “culturally sensitive services” in relation to religious faith and spirituality can lead to a denial of the issues of attempted suicide and self harm’ (Batsleer *et al*. 2003: 110). Understanding diversity of belief within a BME community is highlighted in the research on Chinese people, ‘ethnic minority groups have diverse ways of connecting their lifestyles to their heritage and … their cultural beliefs are not monolithic’ (Chau and Yu 2009: 775).

## Self‐organization as ‘Filling the Gap’

When viewed from an asset‐based approach (Kretzmann and McKnight 1993), it is apparent that people and communities who have found traditional, mainstream services inappropriate or problematic to engage with, can be instrumental in finding appropriate solutions themselves. BME communities have established specific care and support initiatives to address some of the gaps (Manthorpe *et al*. 2010; Truswell 2011; Moriarty 2008; Cant and Taket 2005; Sin 2006). Similarly, LGB people as well as asylum seekers and refugees have found compensatory ways to support themselves through social networks and peer support (Williams 2006; Heaphy *et al*. 2003; Drummond 2002; Papadopoulous *et al*. 2004; White and Cant 2003; Daley 2007). This is especially apparent in the research for older people, carers, dementia and adult mental health. The literature therefore suggests a tradition of self‐organization and informal social support, which is also emerging for micro‐provider activity (Bull and Ashton 2011; Community Catalysts 2011). Self‐organization can encompass a range of different types of mobilization of social and community resources, with narrower or broader understandings of the ‘self’, including people caring for themselves, for friends or for family members and people in ethnic or geographic communities. Definitions of ‘self’ can also correspond to common aims or needs, resulting in self‐organized alliances of different groups for a common purpose (Bystydzienski and Schacht 2001).

The self‐organizing groups in social care and mental health described in this article operated at a small‐scale, frontline or grassroots level. People who ran them were usually from those communities or service user groups and many groups did not seem to be formally constituted (either as businesses or charities) or have formal governance structures (Truswell 2011; Radermacher *et al*. 2011; Bowes 2006). All of the included studies and commentaries suggested that the groups were run on a not‐for‐profit basis or were dependent on grants, often with fragile funding. Most studies suggested that funding was the major issue in the sustainability of the self‐organized support, and others noted the influence of specific managerial skill sets on longevity (Seebohm *et al*. 2013; Radermacher *et al*. 2011; Truswell 2011; Walsh and O'Shea 2008; Bowes 2006).

The research studies suggest that these informal networks and small community services are important for combating isolation and ensuring that older people (including those with dementia) remain supported in rural areas where access to mainstream services can be problematic (Walsh and O'Shea 2008; McDonald and Heath 2008). A study of rural dementia support in Eastern England concluded that, ‘very local services that had grown up to meet particular needs are … celebrated for their sensitivity to older people's sense of security and belonging’ (McDonald and Heath 2008: 17).

Small community‐based or local initiatives have been found to have benefits not only for the users of the service but for the wider community in terms of social inclusion and social cohesion, particularly for isolated older people. One study based in rural Ireland showed the wider impact of an older people's group project providing innovative ‘intergenerational and intercultural projects, drama, health initiatives, life‐long learning, holidays and social events’, as well as more traditional ‘transport, laundry, chiropody, outreach service, information sessions and information technology tutorials’ (Walsh and O'Shea 2008: 797). The intergenerational and intercultural activities were effective in promoting social cohesion and interaction with the local traveller community, many of whom initially became involved through a choral group. The study reflects findings from similar project evaluations on empowerment, reciprocity and compensation (Seebohm *et al*. 2013). Walsh and O'Shea (2008: 802) found:

In an organisation working for a marginalised section of the population, it is noteworthy that empowerment is embedded as a central element of the group's activities … [the project] has succeeded in nurturing a viable social care community … where people do things for themselves and others. This community model compensates for the absence of public provision. Older people make a difference to social care provision and quality of life within [the locality] and its rural environs

However, to balance these findings on social cohesion and inclusion, research into refugee integration and community groups in a densely populated urban area concluded that, ‘adequate resources and social infrastructure for all residents were seen as necessary for cohesion and therefore matching resources and services to community growth is the key to limit the build up of community tension, particularly in areas of existing deprivation and high competition for resources’ (Daley 2007: 168). In other words, such community groups cannot thrive in a context of state withdrawal and resource scarcity.

Compensatory self‐organization is also apparent in the literature for the Chinese community in the UK, for South Asian carers and for LGB older people, but it also shows that cultural assumptions and stereotypes of ‘families’ mean that such self‐organization can be misunderstood or misinterpreted by mainstream services and staff, possibly leading to a lack of appropriate formal support provision (Cronin *et al*. 2011; Price 2012; Heaphy *et al*. 2003; White and Cant 2003; Sin 2006; Yeung and Ng 2010; Katbamna *et al*. 2004; Victor *et al*. 2011).

Most of the grassroots and support networks for BME communities, LGB people, older people living in isolated rural areas and refugees and asylum seekers alike are multidimensional or holistic in their support provision (Cant and Taket 2005; Manthorpe *et al*. 2010; McDonald and Heath 2008; Cant 2002; Cronin *et al*. 2011; Walsh and O'Shea 2008; Drummond 2002; Seebohm *et al*. 2013). Cant describes social support that is beneficial to health and well‐being as being ‘primarily emotional or primarily instrumental … [or] a mixture of both’ (Cant 2002: 1). While mainstream, traditional services tend to focus mainly on instrumental support, emotional and social support was seen as equally important by all groups included in this review and this is prioritized or balanced in the specialist community‐based support projects in the research. One approach to support provision which emerged from the research as being promising for was the self‐help or mutual model (Cant and Taket 2005; Walsh and O'Shea 2008; Seebohm *et al*. 2013). Seebohm *et al*.'s (2013: 398) study of self‐help organizations, including those for BME people, in Essex and Nottingham suggests that:

the groups improved mental well‐being, benefiting individuals and creating community‐based resources. Participants controlled groups activities, gained self‐esteem and knowledge, enhancing scope for self‐determination and choice … Giving was important, helping members to gain a sense of belonging and being involved.

Similar findings come from a study of a user‐led project for older people in rural Ireland and from research into an Irish pensioner's project in London (Walsh and O'Shea 2008; Cant and Taket 2005). The literature in this review has suggested that members of marginalized communities often utilize their own social network resources for support, and the grassroots mutual approach can build on this tendency and recognize it as an asset.

Another dimension to small or specialist community support is that it also contrasts with the mainstream by integrating non‐conventional, informal and broader support sources for individuals and communities. This was a theme coming from several studies, particularly for those focusing on projects for older people living in rural areas and for those from BME communities (Walsh and O'Shea 2008; McDonald and Heath 2008; Manthorpe *et al*. 2010). Research into the development of services for people living with dementia in a rural area concluded that, ‘examining [very local] services in rural areas attenuates many aspects of providing person‐centred care … it requires a whole‐systems approach to service, which includes looking at transport and leisure services as well as at health and social care and how they interact within a community’ (McDonald and Heath 2008: 17). Similarly, Manthorpe *et al*. (2010: 34) located effective mental well‐being activity for older people from BME communities not within traditional mental health services, but within ‘voluntary and community groups, sheltered housing, day care and care management’. Sin (2006) argues that for Asian‐Indian older people living in Britain there needs to be a greater understanding of ‘the interdependence of formal and informal spheres’ of support. A study into the relationship between unmet needs, social networks and quality of life of people living with dementia at home found that while most had ‘physical and environmental needs met by services, psychological and social needs were more likely to be met by those with higher community‐involvement social networks’ (Miranda‐Castillo *et al*. 2010: 1).

The research showed that informal support is not restricted to people or networks providing ‘care’ (Cant 2002; Manthorpe *et al*. 2010). For some BME people, including refugees and asylum seekers, faith, belief and cultural tradition can be a positive factor for resilience, well‐being and for interpreting illness or disability (Mir and Tovey 2003; Papadopoulous *et al*. 2004). Maintaining links to community of culture and identity was also found to be important for the quality of life and well‐being of older LGB people, with research indicating that, ‘lesbian and gay groups or communities [are] important sources of support and provided a means of maintaining a gay or lesbian identity and way of living’ (Price 2012: 525). For older Somali men at risk of depression, social gatherings and reminiscence were important for reducing isolation and promoting mental health, ‘such practices, based on shared cultural, religious and moral values contributed to a strong sense of personal identity’ (Silveira and Allebeck 2001: 313). Specialist, community‐based initiatives appear to be important for ‘offering a place where patterns of cultural specificity [are] part of the everyday life of the project’ (Cant and Taket 2005: 265).

## Intersections between Mainstream and Specialist Support – Bridging the Gap?

Whilst self‐organization offers a pragmatic response to the perceived limitations of mainstream support, it is clear that such activity and provision needs capacity building, funding and infrastructure while maintaining its uniquely responsive, local, grassroots nature. A consistent theme coming from the literature was the need for traditional and mainstream social care and support to understand and accommodate the sometimes complex nature of informal support for BME communities, refugees and asylum seekers and LGB people (Merrell *et al*. 2006; Sin 2006; Victor *et al*. 2011; White and Cant 2003; Heaphy *et al*. 2003). Many of the studies reflected on the relationship dynamic between large mainstream, generalist services (either in the statutory or charity sector) and small, local specialist and community support activity, particularly for BME communities and refugees and asylum seekers. The recommendation in some of the research about the need to explore and develop partnerships in order to develop capacity was qualified with a warning about maintaining the uniqueness of small specialist and community organizations, particularly as regards cultural intelligence and values (Truswell 2011; Radermacher *et al*. 2011; Seebohm *et al*. 2013; Drummond 2002). This was especially apparent in a paper on BME and refugee communities and the implementation of the UK National Dementia Strategy in London for what are described as ‘fourth sector organisations’ (Truswell 2011: 117):

… very small and highly specialised voluntary organisations … with substantial highly specialised skills and information. These organisations required the support of the larger third sector to gain funding, but risked losing their specialised skills and unique contributions if they were permanently absorbed into larger organisations

The current service system favours big organizations with larger capacities and yet because of their scale such services may not have the ‘specific and unique skills and experiences’ of the small, community‐based support organizations for older BME people (Radermacher *et al*. 2011: 558). Similar concerns about small, local self‐help initiatives partnering with large national charities arose from research into specialist self‐help/mutual aid projects in England, ‘Eight groups were affiliated to national charities to get support and status. Relationships varied from inspirational to indifferent or worse for three groups who found that their national charity failed to appreciate the group's voluntary ethos’ (Seebohm *et al*. 2013: 398).

The literature shows that there are issues about capacity building, funding, sustainability and infrastructure for small local, often specialist organizations and projects for people marginalized in mainstream adult social care and support provision (Truswell 2011; Walsh and O'Shea 2008; Radermacher *et al*. 2011; Bowes 2006; Seebohm *et al*. 2013). Some of the difficulties are inherent in the ‘top–down’ partnership dynamics with large mainstream agencies and national charities, as discussed above. The research showed that for small organizations in the BME and rural voluntary and community sector, groups are often competing with each other for funding and lack resources or lack access to funding owing to capacity and skills (Truswell 2011; Walsh and O'Shea 2008; Bowes 2006). This then impacts on the sustainability and capacity of the organizations and their projects.

The ability to apply for and access local authority or health funding related to constitutional issues, skills, time, structure and size along with funding application processes and requirements. This was found to be a particular difficulty for specialist self‐help groups, ‘many groups wanted assistance with fund‐raising and practical matters, especially where there was no specialist support for self‐help groups’ (Seebohm *et al*. 2013: 398). Radermacher *et al*. (2011: 555) note that, ‘smaller organisations reported they were primarily occupied with direct service delivery and administrative tasks’, which limited their capacity for partnerships and fundraising and risked inequalities in partnership working. The study concluded that, ‘the current service system favours bigger organisations with larger capacities’. Bowes outlines the tensions experienced by South‐Asian community‐based groups, where the organizations, ‘demonstrate on one hand that responsive services can be developed at local level, but that on the other, such groups experience exclusion from the general service provision systems. Marginal funding, insecurity and reduced regulation all served to restrict the potential of these groups’ (Bowes 2006: 751). Watters and Ingleby (2004: 556) found similar conclusions for UK refugee and asylum seeker support which they argue is often ad hoc and short‐term, ‘a further problem for both black and minority ethnic groups and refugees is that specialised services targeting these groups often take the form of short‐term projects’.

Mainstream administrative processes and regulation were found to be a potentially restrictive factors for the sustainability of small, specialist community‐based social care initiatives (Bowes 2006; Bernard 2005; Seebohm *et al*. 2013). Bowes demonstrates this for South‐Asian community groups where, ‘there was evidence that they were not subject to the same monitoring and inspection as other contracted‐out service providers, and, at least partly because of this, were not receiving the support local authorities could have offered them’ (Bowes 2006: 751). One of the few evaluations of an innovative social care and support approach looked at recruitment and retention of carers in adult placement schemes in England. While adult placement schemes attracted higher numbers of carers from South Asian backgrounds than in the general social care workforce, in general the ‘most common reason for recruitment difficulties experienced by the schemes was the burden of regulatory requirements and was cited … as the main reason for retention problems’ (Bernard 2005: 566).

While the research shows tensions between larger, traditional mainstream provision and smaller, specialist community support initiatives, it also demonstrates the value of these small organizations for improving mainstream services if the gap can be bridged (Mir and Tovey 2003; Yeung and Ng 2010; Papadopoulous *et al*. 2004; Merrell *et al*. 2006; Cant and Taket 2005; Manthorpe *et al*. 2010; Moriarty 2008). Studies suggest that user and carer groups can be instrumental in providing support for people from South‐Asian and Chinese communities to be aware of available mainstream services and to influence how that support meets the needs of the particular group (Mir and Tovey 2003; Yeung and Ng 2010). A study of the health and social care experiences of Ethiopian refugees and migrants in the UK showed that, ‘participants reported Ethiopian community organisations often played a crucial role in advocating for them and helping them access statutory services’ (Papadopoulous *et al*. 2004: 64).

UK adult social care policy determines that people should have choice and control over their care and support, which clearly implies social care market diversification and involvement in local planning and commissioning (IPC 2012). Having a range of support and advice services appropriate for local populations is important for achieving this, especially where those populations are diverse or if there is a high density of BME or LGB people or refugee and asylum seekers living in the locality. However, research here suggests that mainstream, traditional services should not abdicate responsibility for these groups to specialist services alone (Bowes 2006). While some are very supportive of the idea of specialist services, other LGB older people have expressed anxiety about ‘ghettoization’ if specialist services are their only choice and mainstream support continues to be inaccessible (Price 2012). Bowes (2006: 753) concluded that, ‘while … community‐based South Asian groups provided important and effective services … they do not necessarily represent all those who need service support’. A research review of the health and social care experiences of BME older people found that although ‘people from BME groups reported better experiences from services that specialised in supporting people form minority ethnic groups … if commissioners relied only upon specialist services, this could discourage them from making improvements to mainstream services’ (Moriarty 2008: 5).

## Conclusion and Recommendations

In order to explore the extent of micro‐provision for an increasingly diverse society, this article examined recent research on how certain groups with ‘protected characteristics’ under the Equality Act 2010 (or seldom heard groups) have experienced mainstream and small‐scale provision. Overall, the research evidence on the effectiveness of local community, specialist or small‐scale services is patchy but indicates that information, care and support initiatives are developing in response to actual or perceived difficulties with mainstream provision. There were very few specific service evaluations and none explicitly on small private or not for profit micro‐providers or social enterprises.

Research about BME people, LGB people, refugees and asylum seekers and people from faith communities was identified and examined to discover how local community, specialist or small‐scale services are responding to unmet need for support and advice among these seldom heard groups. While each group had their own particular issues, there were common experiences and responses, most notably self‐organization and mobilization of social capital to compensate for gaps in mainstream support provision. Personalization policy in adult social care is explicit about increasing choice and control over care and support and about building community capacity as part of this (TLAP 2011). While this review article is limited and could not include all seldom heard groups (partly because research could not be identified), the findings reveal some important recommendations for micro‐providers, local social enterprise and commissioners working to achieve personalization and build community capacity for seldom heard groups.

There is a tradition of compensatory self‐organization, use of informal networks and a mobilization of social capital for all these groups in response to marginalization from mainstream, statutory services. This requires recognition and nurturing in ways that do not stifle its unique nature. Marginalization is characterized by fear of discrimination and loss of control, experience of inappropriate support, concern about stigma and communication difficulties. Specialist and community‐based micro‐providers can contribute to a wider range of choices for people who feel larger, mainstream services are not suitable or accessible. However, the types of compensatory activity identified in the research need recognition and investment, and its existence does not imply that the mainstream should not address marginalization. A study of the social care and support expectations of Asian‐Indian older people suggested that, ‘it must … not be assumed that simply because someone is not accessing state support at the moment that the individual thinks that the state does not need to provide these sources of support’ (Sin 2006: 222).

Thus micro‐provision needs to be understood not only as way to fill the gap left by mainstream services, but also as a way to bridge that gap, and make mainstream services more aware of the support needs of diverse communities and particular populations. Local specialist and community support organizations can offer cultural intelligence, opportunities for self‐help/mutual support, a collective voice, social and emotional support and broader and more holistic understandings of support. Such organizations need investment in terms of funding but also capacity building and skills development for sustainability. Processes (including those for commissioning and funding) and regulation need to be proportionate and accessible for small community‐based providers. Large mainstream services should not abdicate responsibility for providing culturally sensitive, accessible support to local specialist and community organizations. However, there are potential opportunities for shared learning and development between the two. Specialist and community organizations can help seldom heard people engage with mainstream services and reduce stigma.
